# Developing young men’s wellbeing through community and school-based programs: A systematic review

**DOI:** 10.1371/journal.pone.0216955

**Published:** 2019-05-20

**Authors:** Kate Gwyther, Ray Swann, Kate Casey, Rosemary Purcell, Simon M. Rice

**Affiliations:** 1 Orygen, the National Centre of Excellence in Youth Mental Health, Melbourne, Victoria, Australia; 2 Centre for Youth Mental Health, the University of Melbourne, Victoria, Australia; 3 Crowther Centre, Brighton Grammar School, Melbourne, Victoria, Australia; 4 Youth Mood Clinic, Orygen Youth Health, Melbourne, Victoria, Australia; Jagiellonian University Medical College, POLAND

## Abstract

Boys and young men have unique health-related needs that may be poorly met by existing programs and initiatives. The mismatch between the needs of boys and young men and current service offerings–driven largely by social determinants of health such as masculinity–may stymie health status. This is evidenced through high rates of self-stigma, accidental death or suicide, and low rates of help seeking and health literacy among populations of boys and young men. With growing interest in improving wellbeing and educational outcomes for all young people (including boys and young men), this systematic review aimed to evaluate community and school-based programs with specific focus on program features and outcomes directly relevant to young males aged 12–25 years. Five data-bases were searched; Medline, EMBASE, PsycInfo, ERIC, and ERAD. Articles were included if they evaluated an intervention or program with a general or at-risk sample of young men, and measured a psychological, psychosocial, masculinity, or educational outcome. The majority of the 40 included studies had high quality reporting (62.5%). Synthesised data included theoretical frameworks, intervention characteristics, outcomes, and key results. Of the included studies, 14 were male-focussed programs, with masculinity approaches directed towards program aims and content information. The emergent trend indicated that male-targeted interventions may be more beneficial for young men than gender-neutral programs, however, none of these studies incorporated masculine-specific theory as an overarching framework. Furthermore, only three studies measured masculine-specific variables. Studies were limited by a lack of replication and program refinement approaches. It is concluded that there is significant scope for further development of community and school-based health promotion programs that target young men through incorporation of frameworks that consider the impact of gendered social and environmental determinants of health. Evaluation of these programs will provide researchers and practitioners with the capacity for translating beneficial outcomes into best-practice policy.

## Introduction

Young men exhibit distinct health and service engagement profiles from that of their female and adult male counterparts [1]. It is widely known that young men are at elevated risk of perpetrating and experiencing aggression or violence, and have higher rates of conduct disorder, accidental death, and suicide comparative to young women and adult males [2–4], yet targeted programs supporting young men’s access to, and engagement with services to support their health and adaptive behaviour are lacking [5]. Differences in the needs of young males are also evident through the inconsistency between self-reported wellbeing and health statistics. It is common for young men to report better subjective wellbeing and satisfaction with life [6, 7], despite indicators of ill-health being higher in young men than young women [3, 8, 9]. Over the past 20 years, the number of deaths from intentional self-harm in youth aged 15–24 has been frequently estimated as two–three times higher in males compared to females [10–12].

Social determinants of health, in particular masculinity [13], are important in understanding young men’s health status and health-related behaviours. Masculinity is shaped by societal expectations, values, and behaviours deemed essential of a ‘man’ [14]; as boys develop into young men these social pressures of ‘being a man’ can assert both positive and negative influences on their self-development [15]. The usefulness and broader societal value of adherence to inflexible notions of masculinity among boys and young men has been widely critiqued, especially from an educational perspective [16, 17]. Extremely gender-typed boys and girls have reported lower levels of school engagement than their less gender-typed peers [18], and there is evidence that boys emotional stoicism behaviours in friendships are associated with lower academic achievement [19]. For adolescent males, denial of vulnerability and emotional or physical control, in addition to risk-taking activities are key gendered norms that shape behaviours and attitudes [20]. As the extant literature highlights the association between conformity to certain male role norms and men’s health related problems and help-seeking [21], it is imperative that health promotion programs can effectively engage boys and young men. Due to differing health profiles and social influences, interventions and health promotion programs may resonate differently according to gender. It stands to reason then, that interventions specifically addressing, or incorporating masculinity-based factors, may have greater acceptability, engagement, and impact with populations of boys and young men [22]. Such approaches include community-based rite of passage experiences that seek to foster healthy identity development and maturity [23, 24], and sports-based approaches leveraging aspects of masculinity, as well as key role models and influencers [25].

In the health promotion field, previous systematic reviews have investigated mental health and intimate partner violence prevention programs in mixed-gender adolescent and young-adult samples, reporting improved outcomes from group-based and experiential programs that focus on health promotion across both community and school settings [26, 27]. Reviews that have examined males in particular have generally focussed on related health behaviours and help-seeking [28, 29], or interventions for sexual and reproductive health behaviours [30, 31]. These male-specific reviews conclude that common barriers to help-seeking are aligned with themes of masculinity (e.g., difficulties showing vulnerability), and masculine-focussed health interventions were identified as more effective than programs without a male approach [28–30]. Further, a recent scoping review for mental health promotion programs with adult male samples (or mixed-gender samples with disaggregated data) found that 22 of 25 studies reported significant positive changes in men’s mental wellbeing [32].

There are currently no published systematic reviews that have specifically investigated engagement with health and positive identity promotion programs for young males that are community and/or school-based. Given schools are increasingly viewed as venues for such initiatives [33], especially for externalising problems such as aggression that are experienced primarily by males [34], this focus was seen as important. Moreover, the broader inclusion of community programs allows investigation of ‘at-risk’ or underserved young men who may have disengaged from school. The primary aim of this review was to identify community and school-based programs in young male samples (or gender-disaggregated samples), with an intent to appraise potential effectiveness of gender-focussed and non-gender focussed programming.

## Methods

### Literature search

A systematic search of five psychological, medical, and education databases (Medline, EMBASE, PsycInfo, ERIC, and ERAD) was conducted for all articles up to September 2018, with the advice of a research librarian. The keywords used for searching can be found in Table 1. Searches were conducted using the combinations 1 AND 2 AND 3 AND 4, though the small number of sourced articles warranted secondary searches using the combinations 1 AND 2 AND 3 (see Table 1). Data bases with MeSH capabilities were additionally searched using the combination Adolescent/ AND Health Promotion/ AND Masculinity/ AND (Male* or Men or Boy*). Researchers also manually searched Google Scholar and relevant references within sourced articles.

**Table 1:** Search terms by grouping construct.

1. Intervention	2. Health	3. Young Men	4. Masculinity
Interven*	Health	Young	Masculin*
Program*	Wellbeing	Adolescen*	Male
Prevention	**adj2**	Teen*	**adj2**
Initiative	Mental	**adj2**	Role*
Strateg*	General	Man	Norm*
Training	Promot*	Men	Attitud*
Educat*	Literacy	Male*	Ideolog*
Teach*	Fitness	Boy*	Behavio*
Course			Identit*
			Conform*
			Hegemon*
			Toxic

*Indicates truncation.

### Study inclusion

Two authors (KG and SMR) independently reviewed eligibility of the sourced records based on the title and abstract. Studies included were based on the following inclusion criteria: (a) male sample or gender data analysed and reported separately for male and female samples; (b) mean age between 12–25 years at the beginning of intervention; (c) implementation of an intervention or health promotion program (all study designs excluding case studies eligible); (d) psychological, psychosocial, masculinity, help-seeking or educational outcomes measured; (e) general or at-risk samples (e.g., samples with subthreshold psychological disorder symptoms, or school samples with students at-risk of academic disengagement). Studies excluded were based on the following exclusion criteria: (a) all female samples; (b) studies focussing on youth offenders, clinical or out-patient samples; (c) case studies; (d) biological, medical or supplementary (e.g., dietary supplementation) interventions; (e) outcome variables relating to reproductive health behaviours (e.g., condom use), partner violence, substance use, physical health (e.g., BMI), smoking and program feedback only. Authors collaboratively discussed and agreed upon inclusion of any studies where application of the inclusion and exclusion criteria was unclear. This systematic review was conducted in accordance with the Preferred Reporting Items for Systematic Reviews and Meta-Analyses (PRISMA) guidelines (see S1 Material for PRISMA checklist [35]).

### Data extraction

Two authors (SMR and KG) designed a standardised data extraction template. KG sourced relevant information including study design, intervention type, intervention setting, theoretical framework, masculinity focus, outcome measures, sample characteristics, assessment schedule, and key results; SMR reviewed data for consistency. Masculinity focus was evaluated using the World Health Organisation’s classifications of *gender-transformative*, *gender-sensitive*, and *gender-neutral* health programs [30]. An intervention was considered to be *gender-transformative* if the program aimed to rework maladaptive male gender roles and promote gender equitable relationships. *Gender-sensitive* programs were those that recognised the specific needs of males in response to socialised gender roles, specifically tailoring program information to young men. *Gender-neutral* programs did not incorporate any gender-focussed aims or tailored information. The authors conducted a narrative synthesis of the results due to widespread variation among intervention types, settings, foci, and outcomes which prohibited a meaningful meta-analysis.

### Quality appraisal

Two established quality appraisal tools were used, the Joanna Briggs Institute (JBI) Critical Appraisal Checklist for Quasi-Experimental Studies, and the JBI Critical Appraisal Checklist for Randomized Controlled Trials (RCTs)[36]. The appraisal score represents the proportion of ‘yes’ responses out of the total number of criteria. ‘Not reported’ (denoted as ‘?’) was treated as a ‘no’ response. If a criterion was not applicable (‘N/A’) to a given study, that item was not counted in the total number of criteria. See S3 Table for full appraisal.

## Results

### Literature search

**Fig 1:**
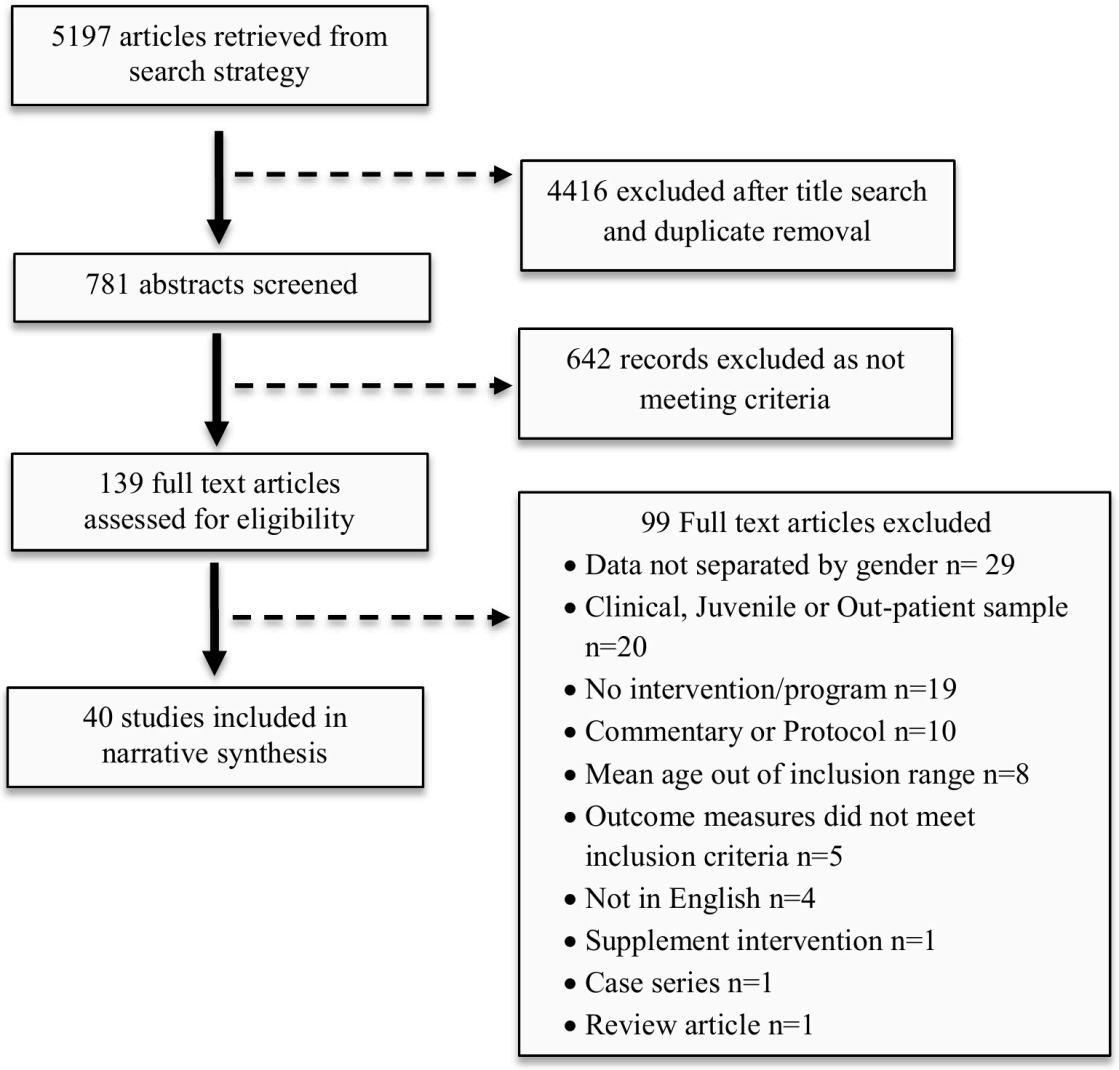
PRISMA flow diagram.

The literature search returned 5197 articles. After removal of duplicates and screening of titles and abstracts, 139 full texts were assessed for eligibility. A total of 40 studies met the inclusion criteria and were assessed in the final review (see Fig 1 for full search flow). Table 2 provides a list of the included studies, categorised by gender focus.

**Table 2:** Included articles categorised by intervention gender-focus.

**Gender transformative**	**Gender neutral**	
Edwards, van de Mortel & Stevens, 2017 [37]	Bademci, Karadayi & de Zulueta 2015 [38]	Opper et al. 2014 [39]
Liddell & Kurpius, 2014* [40]	Bannink et al. 2014 [41, 42]	Rhodes et al. 2008 [43]
Namy et al. 2015 [44]	Bluth, Robertson & Girdler, 2017 [45]	Ritchie et al. 2014 [46]
Smith 2012 [47]	Campbell-heider, Tuttle & Knapp 2009 [48]	Rojiani et al. 2017 [49]
**Gender sensitive**	Castillo et al. 2013 [50]	Sekizaki et al. 2017 [51]
Ashton et al. 2017 [52]	Crooks et al. 2017 [53]	Shoshani & Steinmetz 2014 [54]
Broadbent & Papadopoulos 2014 [55]	Eather, Morgan & Lubans 2016 [56]	Sibinga et al. 2013 [57]
Burns et al. 2010 [58]	Eteokleous 2011 [59]	Skre et al. 2013 [60]
Lubans et al. 2015 [61]	Fuller et al. 2013 [62]	Switzer et al. 1995 [63]
Lubans et al. 2016 [64]	Garaigordobil & Pena-Sarrionandia 2015 [65]	Taylor, Gillies & Ashman 2009 [66]
Marsh & Richards 1988 [67]	García-López & Gutiérrez 2015 [68]	
McCabe, Ricciardelli & Karantzas 2010 [69]	Kerr, Burke & McKeon 2011 [70]	
Shandley et al. 2010 [71]	Margalit & Ben-Ari 2014 [72]	
Stanford & McCabe 2005 [73]	O’Dea & Abraham 2000 [74]	
Wade et al. 2018 [75]	O’Kearney et al. 2006 [76]	

*Dissertation

### Study characteristics

For a summary of article characteristics, see Table 3. A detailed description of extracted data for each article can be found in S1 Table. The included studies ranged in date of publication from 1988 to 2018. A total of 34 (85%) studies were published in 2009 onwards, with 22 (55%) studies published in 2014 onwards. Interventions were delivered in 15 countries, with the majority of studies located in Australia (n = 15, 37.5%), followed by the United States (n = 8; 20%; see S1 Table). Of the 40 studies included in the review, four evaluated gender-transformative programs, 10 articles assessed seven unique gender-sensitive programs, and 26 articles evaluated gender-neutral program effectiveness in young men (eight all male samples, 18 with sex disaggregated data).

**Table 3:** Summarised article characteristics.

**Sample Characteristics**		**Intervention Types**	**n (%)**
Mean sample size (n)	237	Physical activity & sport	8 (20%)
Median sample size (n)	96	eHealth & online gaming	7 (17.5%)
Mean age of samples (yr)	15	Psychoeducation	5 (12.5%)
Median age of samples (yr)	15.5	Mentoring	4 (10%)
Aggregate sample size (n)	8,290	Outdoor adventure	4 (10%)
**Masculinity focus**	**n (%)**	Male identity development	4 (10%)
Gender-transformative	4 (10%)	Mindfulness & meditation	3 (7.5%)
Gender-sensitive	10 (25%)	Body-image & self-esteem	3 (7.5%)
Gender-neutral	26 (65%)	Emotional intelligence	2 (5%)
**Study design**		**Intervention Setting**	
Single-group pre-post	11 (27.5%)	Secondary school^a^	28 (70%)
Randomised control trail (RCT)	8 (20%)	Community	6 (15%)
Quasi-experimental	6 (15%)	Mixed	4 (10%)
Experimental	6 (15%)	University	1 (2.5%)
Non-randomised control trial	4 (10%)	Online	1 (2.5%)
Cross-sectional	4 (10%)		
Longitudinal	1 (2.5%)		

^a^14% of secondary school interventions were delivered by trained school staff.

### Intervention characteristics

Intervention types and settings are listed in Table 3. The length of interventions ranged from 90 minutes, to one year. The mean length of intervention was 18 weeks. Half of the programs consisted of 45–90 minute weekly sessions (*n* = 20, 50%). Data were collected pre and post intervention in 35 of 40 articles (87.5%). Follow-up data were collected in 16 of 40 articles (40%), with the length of follow-up spanning from two months to two years after the intervention or program. The mean length of follow-up was 8.6 months.

### Gender-transformative programs

#### Intervention type, focus and setting

The focus for all four gender-transformative programs was the development of healthy masculine identity [37, 40, 44, 47]. Specifically, the ‘Rock and Water Program’ (RWP) focussed on challenging masculine stereotypes of aggression by linking physical exercises to mental and social skills [37], ‘The Council for Boys and Young Men’ (The Council) intervention aimed to encourage solidarity amongst young men, question maladaptive stereotypes, and recognise strengths and collective responsibilities [40], the ‘Young Men Initiative’ (YMI) provoked critical reflection of gendered norms and the impact of gender discrimination to reshape what it means to ‘become a man’ [44], lastly, ‘The Rite Journey’ (TRJ) program implemented traditional ‘rite of passage’ notions–separation from community, learning, and return to community–to mark the transition from boyhood to manhood [47]. Each program consisted of multiple components, including psychoeducation, outdoor adventure, physical activities, team-based games, and collaborative discussion. All four programs were delivered to high school populations, and ran for a minimum of nine weeks up to one school year, with an average duration of 30.8 weeks. The Council and RWP consisted of 90-minute weekly sessions, and TRJ and YMI was delivered in 60-minute weekly sessions with the addition of an outdoor camp. Programs were generally led by external trainers, whereas TRJ was delivered by school staff. No articles reported follow-up data.

#### Theoretical frameworks

Studies reporting results for The Council, YMI, and TRJ reported relevant theoretical frameworks for their respective programs. The Council was based upon resiliency principles and relational-cultural theory, focussing on growth and development that occurs through connection, mutual empathy, and empowerment [77]. Peer-group learning and socialisation framed the YMI program, and TRJ was built on the rites of passage ‘five-c’s’ model of consciousness, connection, communication, celebration, and challenge. The importance of growth and learning through connection with others was a common theme across frameworks.

#### Key results

Each of the four gender-transformative interventions reported some positive changes in participants post-program. The Council was the only gender-transformative program to report quantitative outcomes, with significant positive intervention effects observed for school self-efficacy and future self-efficacy. Notably, no changes were found for masculine ideology, relational aggression or identity distress [40]. The YMI, TRJ, and RWP reported qualitative outcomes. Comments from participants suggested that the programs were effective for reducing anger [37], increasing self-reflection, and reshaping perceptions about ‘being a man’ [44, 47]. Of note, the number of TRJ participants that reported changes in their concepts of masculinity (*n* = 3) was equal to the number that reported no changes.

### Gender-sensitive programs

#### Intervention type, focus and setting

There were 10 articles evaluating gender-sensitive programs, of which seven were unique interventions. Four articles assessed physical activity interventions, with three evaluating the ‘Active Teen Leaders Avoiding Screen Time’ (ATLAS) program [61, 64, 75], and one article presenting the ‘Harnessing EHealth to enhance Young men’s Mental health, Activity and Nutrition’ (HEYMAN) intervention [52]. HEYMAN was deemed gender-sensitive as it conducted formative research with young men to develop the program around their preferences. ATLAS targeted young males through discussion and development of strength and muscular fitness, and their links to self-esteem among boys [78]. Similarly, two studies assessed body-image promotion programs [69, 73], The ‘Healthy Body Image Program’ (HBIP) targeted concepts related to male body-image concern, namely, low self-esteem and poor peer relationships [69]. The second program reported by Stanford and McCabe addressed common stereotypes associated with the ‘ideal’ male body, and prompted reflection around how and from where these notions derived [73].

Two papers evaluated one eHealth intervention, ‘Reach Out Central’ (ROC) [58, 71], an online game to educate and promote mental health. ROC is a gender-sensitive iteration of ‘Reach Out’ in response to young men’s lack of engagement and use of the original Reach Out online service. One psychoeducation program, ‘Incolink Life Skills Programme’, (ILSP) incorporated male-specific mental health, suicide prevention, and outreach information for young men in the building and construction industry [55]. Lastly, the ‘Outward Bound Bridging Couse’ (OBBC) was a residential outdoor program, designed specifically for low-achieving high-school males to encourage understanding of personal strengths, abilities, and motivations, with a particular focus on academia [67]. Gender-sensitive programs were on average 9.3 weeks long, with a minimum length of 90 minutes and a maximum duration of 20 weeks. Programs were delivered in 1–2 hour weekly sessions in six of the 10 studies. ILSP was one 90-minute session, OBBC was a six-week residential program, and ROC had unspecified intensity due to the online nature of the intervention. Half of the interventions reported follow-up data [55, 58, 69, 71, 73], with a mean follow-up length of 4.2 months. Over half (*n* = 6) of the gender-sensitive interventions were implemented in high-schools, or with groups of students [61, 64, 67, 69, 73, 75]. Combinations of school staff and external personnel delivered the intervention content. HEYMAN and ROC were both community-based interventions with online components, and ILSP was implemented in the workplace.

#### Theoretical frameworks

Self-determination theory (SDT) was the foundation of both physical activity interventions (ATLAS, HEYMAN), which posits that satisfaction with three basic psychological needs of autonomy, relatedness, and competence is associated with self-driven motivation [79]. Achievement motivation theory guided development of OBBC, which used principles of learning, social groups, and goal-orientated action to increase motivation [67]. Bandura’s social cognitive theory (SCT) provided a framework for ROC and HEYMAN. SCT postulates that self-efficacy and perceived collective-efficacy influence motivation to perform a behaviour [80]. ROC also integrated principles of Cognitive Behaviour Therapy (CBT) into the online game’s story lines [58, 71]. Articles assessing the two body-image programs and ILSP did not report integration of specific theoretical frameworks [55, 69, 73]. Common concepts across frameworks for these gender-specific programs included motivation and socialisation.

#### Key results

All seven male-specific interventions reported at least one beneficial outcome in young men. Post-program, participants of the physical activity interventions, HEYMAN and ATLAS, reported increased quality of life enjoyment and satisfaction, and psychological wellbeing, respectively [52, 64]. For the body-image programs, HBIP participants with initial body dissatisfaction showed a significant reduction in negative affect post-program [69]. Stanford and McCabe’s body-image program also reported decreased negative affect, as well as increased self-esteem in participants [73]. Self-esteem was also significantly improved after the OBBC, both in the overall and academic domains [67]. Positive outcomes for the ROC and Incolink programs were observed in help-seeking outcomes. Males reported increased likelihood to seek help from a mental health professional following use of ROC, and approximately 80% of participants from the Incolink intervention indicated that the workshop helped them understand how to identify and seek help for problems in themselves and others.

### Gender-neutral programs

#### Intervention type, focus and setting

For the 26 articles assessing programs without a specific male or masculinity focus, the most common type of intervention was mentoring or community service (*n* = 4; [38, 43, 53, 63]), psychoeducation (*n* = 4; [48, 54, 60, 70]), physical activity or sport (*n* = 4; [56, 62, 66, 68]), and eHealth interventions (*n* = 5, 4 unique programs; [41, 42, 51, 59, 76]). There were three outdoor adventure programs [39, 46, 72], three mindfulness and meditation programs [45, 49, 57], two emotional intelligence interventions [50, 65], and one body-image program [74]. For samples of high-school students (*n* = 18, 69%), seven programs were delivered in all boys schools (39%), and 11 in coeducational schools (61%). Programs were generally delivered by outsourced trainers or using external platforms (i.e., eHealth). The shortest program was 90 minutes long and the longest program ran for one year, with an average duration of 14 weeks. Typically, programs were implemented for 1–2 hours per week (*n* = 14; 54%). Shorter intensities included a 2-hour psychoeducation program [70], one 45-minute eHealth program [41, 42], and a one-hour fortnightly psychoeducation program [54]. Outdoor adventure programs had longer continuous intensities, with programs running for four days [72], 10 days [46], and 23 days [39]. Follow-up data was analysed in 11 articles (42%; [39, 43, 46, 48, 53, 54, 57, 60, 65, 72, 74, 76]), with a mean follow-up length of 10.8 months.

#### Theoretical frameworks

Frameworks typically reflected the intervention type, for instance, the emotional ability model was the basis for both emotional intelligence programs. The experiential learning framework was used for the outdoor adventure programs, positive psychology models provided the framework for two psychoeducation programs and one mindfulness program. CBT principles were integrated into one psychoeducational and one eHealth intervention. Singularly implemented frameworks included social-learning theory, hopelessness theory, attachment theory, cooperative learning theory and emotional regulation principles.

#### Key results

There were nine gender-neutral programs that investigated outcomes in male-only samples, of which eight (89%) reported positive effects in young men for at least one outcome. Of these, two outdoor adventure programs reported significant increases in self-efficacy and cognitive autonomy [72], and emotional intelligence, intrapersonal skills, adaptability, and mood [39]. Of the two sporting programs reporting positive effects, one showed promising qualitative results where participants reported increased competence [62], and one reported significant quantitative intervention effects for reduced depressive symptoms following the exercise intervention [66]. For the two eHealth interventions with positive effects, one reported significant short-term improvement in depressive symptoms and a long-term intervention effect for self-esteem [76], and one found a preventative effect in distress symptoms whereby the intervention group showed a non-significant decrease and the control group showed a significant increase in distress [51]. One mindfulness intervention reported significant reductions in anxiety symptoms and rumination [57], and one mentoring program conveyed positive qualitative outcomes, with participants self-reporting decreased aggression, and increased motivation [38]. The one intervention that did not show an effect was a two hour psychoeducational session regarding depression [70].

The remaining 17 articles evaluated gender-neutral programs in mixed-gender samples. Of these articles, seven (41%) reported positive changes in mostly males, six (35%) reported intervention effects in mostly females, three (18%) found positive changes in both genders, and one (6%) reported no intervention effects in either gender. The seven programs with benefits in boys included two psychoeducational programs that improved mental health [48], and self-efficacy and optimism [54], one mindfulness and one community service program that reported reduced negative affect [45, 63], an emotion intelligence intervention that increased emotional attention and clarity [65], a culturally-relevant eHealth program that increased interest in diversity of contact [59], and lastly one sport program that increased assertiveness [68]. Programs which were less effective in young men compared to young women included two mentoring programs [43, 53], two psychoeducation programs [41, 42, 60], and one meditation program [49]. Females typically reported higher satisfaction and engagement with the programs [41–43], higher mental health literacy [60], and lower mental health prejudice [41, 42, 60].

### Quality appraisal

The appraisal score (in proportions) for the 40 studies ranged from moderate (0.5) to excellent (1.0). The average appraisal score was 0.85, with the majority of studies reporting high quality (n = 25, 62.5%), where high quality is regarded as ≥ 0.8. Of these studies, nine met all appraisal criteria. There were 15 studies (32.5%) with moderate quality (appraisal score of 0.5–0.79), and none reporting poor quality (<0.5). For the eight RCTs, all articles used true randomisation techniques, compared similar participant groups, measured variables consistently across groups, accounted for incomplete follow-up, and used appropriate statistical analysis. None of the eight RCTs blinded participants, or research staff delivering the interventions. For the remaining 32 studies, control group use (n = 17, 53%), and complete follow-up (n = 22, 76%) were the lowest scoring criteria. Complete quality appraisal information can be found in S3 Table.

## Discussion

The aim of this review was to identify and appraise the potential effectiveness of school and community-based health and wellbeing programs in young men. The articles identified in this review evaluated a range of intervention types, durations, and intensities. On average, interventions were implemented for 18 weeks, typically through 1–2 hour weekly sessions. Overall, findings support the effectiveness of health promotion programming for boys and young men, especially in gender-focussed interventions and school-based environments, which comprised the most frequently used intervention setting. The percentage of programs reporting positive effects in young men is encouraging (100% of gender-sensitive and gender-transformative programs, 69% of gender-neutral programs), suggesting that participation in these programs, despite the varying aims and activities, is likely to be valuable. Nonetheless, a need remains to determine which approaches work best. While heterogeneity in intervention types, foci, and outcomes of studies included in the present review prohibited meta-analysis, this should be considered as a priority as the field develops, and randomised trials and replication studies are undertaken. It was also clear from results that there is growing interest in the health promotion field for boys and young men, as 55% of included studies were published from 2014 onwards. Despite this growing interest, further investigation is needed for the development of a robust evidence base, critical for well-informed recommendations regarding program development and implementation. This review synthesised findings from a high quality pool of literature (62.5% high methodological quality), with a majority of articles reporting controlled methods with multiple assessment points.

An important aspect of this review involved identifying those approaches that incorporated a specific focus on masculinity, a key social determinant of the health of boys and men [81]. Previously, systematic reviews have focussed on understanding the health-behaviours of men and how they relate to help-seeking, finding that poorer mental health literacy and adherence to rigid male norms prevented help-seeking and increased self-stigma [28, 29]. We were not able to locate any reviews that focussed on young male samples, though for adult men, effective programs were typically gender-transformative and based on theoretic models [30–32]. Extending upon these findings, this review has explored masculine and non-masculine focussed approaches, and existing theoretical frameworks in programs supporting the health of young men.

### Incorporating a masculinity focus

In this review, 10 of the 14 articles that incorporated a masculinity focus evaluated gender-sensitive programs, and four evaluated gender-transformative programs. Encouragingly, all four of the gender-transformative programs reported beneficial outcomes in young men across a range of outcomes, including self-efficacy, anger, and perceptions of manhood. The overarching aim of these four programs however was to help young males develop their own healthy masculine identity, and the relative success of these programs in achieving this aim is less clear. Indeed, there is a need to identify and define the determinants of what actually constitutes (and does not constitute) a healthy masculine identity. While this work is beyond the scope of the present study, it is likely essential to furthering research rigour and scholarship in the domains of young men’s health [82]. Participants of ‘The Council’ did not show any quantitative changes in masculine ideology, and an equal number of TRJ participants reported experiencing, and not experiencing, changes to their perceptions of being a man. Nevertheless, these programs reported reduced anger and improved self-efficacy [37, 40], which are likely important aspects of a healthy masculine identity.

Similarly, all ten articles evaluating gender-sensitive interventions also reported positive outcomes in young males. The aims of these interventions were focussed on improving self-esteem, school engagement, physical activity and mental health awareness in young men. The effectiveness of these programs are evidenced by improvements in the outcomes related to intervention aims. For instance, the OBBC had a focus on school engagement and reported increased academic self-esteem in participants. Moreover, the body-image and physical activity interventions found improvements in self-esteem, negative affect, quality of life enjoyment, and psychological wellbeing, all of which are constructs that have been repeatedly linked with positive body-image and physical activity [83, 84]. Lastly, ROC and Incolink both delivered psychoeducational components, and reported high psychological help-seeking intentions in participants post-program.

A number of gender-neutral programs were also effective for health promotion in boys and young men. Notable improvements were found for measures of self-efficacy, competence, negative affect, and depressive symptoms. These benefits were commonly identified following programs with a focus on experiential learning and shared-activity, for instance outdoor adventure, sporting, and exercise interventions. This aligns with men’s preferences for group interaction and informal spaces as facilitators to engaging with mental health services [85]. Despite this, there were also a number of gender-neutral programs that were more effective in young women than young men. Females repeatedly showed stronger program engagement, mental health literacy, and lower mental health stigma compared to males. These findings suggest that young men may be more likely to value programs that incorporate male-specific components, especially in relation to program engagement, rather than gender-neutral interventions. Male-targeted messaging has been identified by young men themselves as a strategy to improve engagement with community mental health services [86, 87]. This extends from delivering information about men’s mental health, to utilizing spaces frequented by young men, such as sporting clubs and specific social media avenues, as locations to deliver tailored health information and intervention [86].

The reported lack of improvement in young men’s mental health stigma may be associated with low program engagement. Stigma has been repeatedly recognised as a key barrier to access and engagement with mental health services for boys and young men [3, 88–91]. Disengagement, or lack of engagement, with services may perpetuate negative attitudes that young men and boys typically hold regarding mental ill-health, such as associated feelings of perceived weakness or shame [3, 92]. If young men do not perceive an intervention as worthwhile, they may generalise this view to other health behaviours such as help-seeking. It is imperative to therefore extend program engagement as this may help to reduce mental health stigma in young men. Nonetheless, synthesis of interventions by gender-transformative, gender-sensitive, and gender-neutral approaches indicates that incorporation of some male-specific approach, whether it is in the core aim of the program or in tailored content information, can have a positive impact on the health and psychological functioning of boys and young men.

### Framework development

Theoretical frameworks supporting the interventions were diverse, with 22 different theories implemented across areas of learning, cognition, motivation, socialisation, and culture. However, common themes of socialisation and connectedness emerged when assessing program frameworks associated with positive outcomes in young men, especially in gender-transformative and gender-sensitive interventions. The health benefit of social support in young men is associated with enhanced wellbeing [93]. Socialisation may also impact masculinity as young men frequently evaluate their male identity against their peers [94, 95], and it is suggested that friendships can provide space for young men to ‘try out’ masculine identities [96]. Programs that integrate social activities may give young men the space to acknowledge the existence of different masculinities amongst those around them, and to feel comfortable in expressing their own male identities.

Unfortunately, the frequency of articles that did not mention any theoretical frameworks (*n* = 7; 17.5%), is problematic for program evaluation and understanding theorised mechanisms of change, and hinders the development and refinement of future health promotion programs for boys and young men. Moreover, none of the male-focussed interventions incorporated masculine-based frameworks, instead citing general psychological frameworks including cognitive behaviour therapy or self-determination theory [64, 71, 75]. This in itself is not necessarily a limitation, though integrating masculinity frameworks could further improve outcomes in young men through focussed targeting of potential mechanisms of change [3], and by extension result in improved societal health.

Relevant masculinity frameworks include Kiselica and Englar-Carlson’s Positive Psychology Positive Masculinity (PPPM) framework [97], and the Health, Illness, Men and Masculinities (HIMM) model [4]. The PPPM framework aligns with health promotion in young men as the model focuses on endorsing male strengths rather than ‘fixing’ problematic behaviours and beliefs. For example, promoting courage through sensible risk-taking rather than reckless behaviour [97]. The model is flexible as it recognises how ideologies are endorsed differently in men of different cultures and ages. Similarly, the HIMM model explores the interaction between socialised masculine ideologies and other social determinants of health, namely: race, status, sexuality, socio-economic status, education, and community among others [4]. The youth specific focus in the HIMM framework targets the socialised celebration of physical risk tasking and the ‘take it like a man’ attitude in young men [4]. Both models recognise that there is no single standard for masculinity and understand the influence of social constructions in the overall endorsement of masculine norms. Future health promotion programs for young men should consider how their programs could incorporate relevant theoretical frameworks and whether this positively influences masculinity and health outcomes.

### School settings

This review found a high percentage of studies evaluating programs in secondary-school settings (n = 28, 70%). Schools are unique environments for program implementation given students experience connection in an established community with corresponding social values. Moreover, secondary students are at a developmental stage where social and self-identity is in a state of rapid development [98]. However, secondary schools also may perpetuate or favour particular aspects of masculine identity via the perceived importance of popularity, being gifted in (hyper-masculine) sports, and acceptance within male peer-groups [99, 100]. It is important to help young males identify that at times, these norms can be restrictive and problematic, reinforcing patterns of dominant male socialisation [21]. Schools may be optimal settings therefore to implement early intervention programs for healthy identity development in young males, at a time where their masculine identity is forming [101, 102].

Schools are also ideal settings for programming around health-related attitudes, as they embrace an interconnected community system including parents, staff, and alumni [103]. Programs for adolescent boys could include these broader support systems in the framework or program activities to increase social support, connectedness, and respect for all. The role and potential impacts of the broader community was seemingly overlooked in reporting of the development and implementation of school-based programs included this review, which may have reflected time-limited programs that were implemented without full support of the school community. For example, school-based programs included in the present review typically invited outsourced groups or research personnel to administer the programs (*n* = 12), with only a handful of trained school staff delivering the program (*n* = 4). Building the capacity and experience of internal school staff to facilitate school-based programs is likely to be an important aspect of program sustainability. Future research should look to identify any barriers and facilitators of program delivery by internal staff.

From this review, it is apparent that there are health promotion programs being delivered to a large number of secondary students without extensive research evaluation. For example, the RWP intervention cited delivery to over 2 million students worldwide [37], though only one evaluation study of RWP fit our inclusion criteria, where authors noted previous assessments of the program were typically anecdotal [37]. Smith [47] commented that TRJ has been applied in Australia for a number of years, though no additional evaluation literature was found in this review. This alerts us to the possibility that other worthy and innovative programs may be widely used, but rarely evaluated. Without proper evaluation, it cannot be determined if such programs are demonstrably effective, or whether they may inadvertently be hindering wellbeing, or perpetuating traditional masculine stereotypes in young men, as opposed to reconstructing or reconfiguring masculine norms [104, 105]. Moreover, without such program evaluation researchers and school bodies will be unable to develop or improve programs to tailor to the needs of their target audience.

### Limitations and future directions

Due to the broad approach taken in this review, the resulting heterogeneity of study characteristics prevented a meta-analysis or assessing publication bias. The possibility of publication bias is considered as most of the 40 included studies reported at least one significant effect, suggesting that studies failing to report an effect may be less likely to be published. Heterogeneity additionally hindered the ability to draw statistical comparisons for specific outcomes, settings, and designs. This review was also limited by the inclusion of studies reported in English only. Review of non-English articles is critical to obtain a comprehensive understanding of the literature, unfortunately we were unable to do so in the scope of this review.

Despite the potential effectiveness of masculine-focussed programming, evaluation of young men’s conformity to traditional masculine norms was limited in this review by the small number of studies incorporating a direct measure of masculinity. It is noteworthy that so few studies seeking to engage boys and young men in attitudes related to masculinity actually sought to measure the construct [106]. There are now at least 16 validated scales to assess masculine ideology, and the Conformity to Masculine Norms Inventory is one of the most widely used measures available in a brief format [107], including use in national population health studies for men [81]. There is a need for further investigation of valid and reliable masculinity-based outcomes in the present research studies. The established link between strict adherence to traditional masculine norms and poorer mental health or related behaviours suggests that altering maladaptive masculine attitudes may improve general wellbeing in young men [4, 28, 108]. It is imperative to better understand how gender-sensitive and gender-transformative programs influence masculine ideologies. Measuring these constructs will additionally allow for deeper analysis between related measures of wellbeing, physical or mental health, or identity development.

Limitations of the included articles and therefore of this review include a lack of long-term intervention, follow-up, and program refinement. The majority of evaluations were conducted for relatively short-term programs that would run once a week for 4–12 weeks. Moreover, it is noteworthy that none of the gender-transformative interventions reported follow-up data. Future studies should collect this data to evaluate sustained or long-term intervention effects. There were also no instances of study replication, though one program was suspected to have been developed from an earlier iteration of the same program [69, 73]. There was also a small subsection of cross-sectional studies that assessed participants up to 12 months after completing very brief programs [55, 70]. The reliability of the outcome measures is reduced if participants are not able to recall the details of the intervention, or when initial effects may have subsided. This pattern of short-term, one-off intervention evaluation results in a lack of effect replication and no evidence of program enhancement. Without repeating evaluations it cannot be determined whether programs are reliably effective.

From this review, we can determine that there is still a large amount of research and program development that needs to occur before researchers have the capacity for translating beneficial outcomes into best-practice policy. Specifically, there is scope for the development of programs directed to young men founded in masculinity frameworks and further quantitative assessment of masculinity variables, such as male-norm adherence and masculine identity-distress, in male-targeted interventions. Alongside this need, documentation of program development should also increase in order to assist future development of similar health promotion programs in young men.

## Conclusion

This review supports the use of community and school-based programs in fostering health, wellbeing, and identity development in boys and young men. Such initiatives are needed in order to provide boys and young men with ‘teaching moments’ to develop necessary skills and attributes they may otherwise not develop. Incorporation of male-targeted approaches through gender-sensitive and gender-transformative programs may also benefit young men’s mental health and wellbeing. There remains a need for research and development of health promotion programs that specifically target young men through incorporation of frameworks that consider, but not necessarily reinforce, gendered social and environmental determinants of health (e.g., masculinity). It is imperative that researchers, program developers, and educators jointly collaborate to strengthen gender-responsive programs that foster healthy lifestyles and wellbeing in young men. Such approaches are likely to positively impact the ways in which boys and young men relate both to others and themselves, and reduce the unnecessary mortality and morbidity associated with boys and young men’s maladaptive behaviours and attitudes.

## Supporting information

S1 Material PRISMA checklist. (DOC)

S1 Table Article characteristics summary. (DOCX)

S2 Table Article results summary. (DOCX)

S3 Table a. Quasi-experimental quality appraisal–Joanna Briggs Assessment; b. RCT quality appraisal–Joanna Briggs Assessment.
(DOCX)
