# Supplementary material for: Developing young men’s wellbeing through community and school-based programs: A systematic review
Source: PLoS One. 2019 May 20;14(5):e0216955. doi: 10.1371/journal.pone.0216955 (PMC6527294; doi:10.1371/journal.pone.0216955)
Supplement: S1 Table — (DOCX) [file pone.0216955.s002.docx]

**S1 Table. Article Characteristics Summary**

| **Authors** | **Study Design** | **Intervention Focus (Name)** | **Intervention Theoretical Framework** | **Intervention setting (Country)** | **Gender / Masculinity Focus** | ***N* (M:F)** | **Mean Age (SD) years** | **Outcome Measures** | **Sample** |
| --- | --- | --- | --- | --- | --- | --- | --- | --- | --- |
| Ashton et al., 2017 | Assessor blinded RCT | Healthy lifestyle program (HEYMAN). Website access, 11x 1hr weekly group physical activity session, 1x 1hr individual physical activity session | Social cognitive theory (SCT) & Self-determination theory (SDT) | Online eHealth & community (Australia) | Yes; Gender-sensitive | 50 (50:0) | 22.1 (2.0) | Satisfaction with life (SWL), distress, quality of life enjoyment | General population |
| Bademci, Karadayi & de Zulueta, 2015 | Single-group; longitudinal | Mentoring, peer-based support (To-Gather with Youth Project; TYP). Weekly mixed activity workshops during a University term | Attachment theory | Centre to support street youth & University centres (Turkey) | No | 30 (30:0) | Range: 14-17 | Quality of life, Self-confidence, school attendance | At-risk; homeless male youth |
| Bannink et al., 2014a, 2014b | 3-armed cluster RCT | Online, personalised health behaviour and wellbeing information (E-health4Uth). 1x 45 minute self-report to receive tailored health information, 1x45 minute self-report & counselling referrals, or inactive control | Unclear (individualised information) | Secondary schools; Online eHealth (Netherlands) | No | 1087 (621:466) | 15.9 (0.7) | Satisfaction and use of program; mental health status, health-related quality of life | General population |
| Bluth, Robertson & Girdler, 2017 | Single-group; pre-post design | Mindfulness intervention (Learning to BREATHE). 6x 1.5hr weekly sessions. Encouraged home-practice | Positive psychology; mindfulness based stress reduction | Community & university (United States of America (USA)) | No | 15 (5:10) | Range: 13-18 | Physiological stress, self-compassion, life satisfaction, perceived stress, positive & negative affect | General population |
| Broadbent & Papadopoulos, 2014 | Single-group; cross-sectional | Informational intervention; mental health, suicide prevention & service outreach (Incolink Life Skills Programme). 1x 1.5hr workshop | NR | Rural & metropolitan construction trade venues (Australia) | Yes; Gender-sensitive | 119 (119:0) | Median 20 | Self-help, peer-support, program feedback | Construction workers |
| Burns et al., 2010; Shandley et al., 2010 | Single-group; pre-post design | Educational life skills single-player video game (Reach out Central; ROC). Plot-based play driven by player-made decisions | Cognitive behaviour therapy (CBT), elaboration likelihood model & SCT | Community (Australia) | Yes; Gender-sensitive | 266 (88:176) | 20.5 (2.1) | SWL, distress, stigma, help-seeking | General population |
| Campbell-heider, Tuttle & Knapp, 2009 | Multi-group; quasi-experimental | Group intervention providing social support, health education and outreach. (Teen Club or Teen Club & Positive Adolescent Life Skills; PALS) 32x weekly group meetings | CBT for PALS | Urban secondary school (USA) | No | 14 (8:6) | Range: 12-15 | Mental health, family & peer relations, social skills, education status, aggression | General population |
| Castillo et al., 2013 | Quasi-experimental with control group | Emotional intelligence (EI) educational program (INTEMO program). 2-year intervention; 12x 1 hr sessions in 1 semester per year | EI ability model | Public middle & high schools (Spain) | No | 590 (269:321) | 13.8 (1.5) | Empathy, aggression | General population |
| Crooks et al., 2017 | Multi-group; pre-post design | Culturally-relevant mentoring program (The Fourth R). 18x 1hr weekly sessions; second group additional one-on-one weekly mentoring sessions over school year | Medicine Wheel life cycles | High schools (Canada) | No | 105 (53:52) | 12.6 (0.6) | Mental health, cultural identity, school climate, SWL | Aboriginal youth; First nations, Métis and Inuit (FNMI) |
| Eather, Morgan & Lubans, 2016 | RCT | Group exercise program (CrossFit^TM^ Teens). 8x 1hr twice-weekly sessions | Multi-dimensional exercise and self-esteem model (EXSEM) | Secondary school (Australia) | No | 96 (46:50) | 15.4 (0.5) | Self-esteem, psychological difficulties | General and at-risk population |
| Edwards, van de Mortel & Stevens, 2017 | Single group; pre-post design | Identity and social development program (Rock and Water Program; RWP). 9x 1.5hr weekly sessions | NR | Rural high schools (Australia) | Yes; Gender-transformative | 187 (187:0) | 13.6 (1.0) | Program engagement & feedback | At-risk; disengaged from school, perpetrators & victims of aggression |
| Eteokleous, 2011 | Single-group; pre-post design | Web-based socio-virtual cultural awareness curriculum (MYTecC). 8hrs/week for 1 year | Multicultural web-based connectedness | Online, universities, community & youth centres (mixed European, Middle Eastern and African) | No | 135 (74:60) | Range: 12-16 | Comfort with differences, universality-diversity orientation & diversity of contact | General population |
| Fuller et al., 2013 | Single group; cross-sectional | Sports-based youth development program (Sport Hartford Boys Program; SHB). 24x 2hr twice-weekly sessions | Five Cs model; competence. confidence, connections, character, caring | Elementary & middle school (USA) | Unclear | 8 (8:0) | Range: 10-13 | Program engagement, social connectedness, self-efficacy | Ethnic minority youth |
| Garaigordobil & Pena-Sarrionandia, 2015 | Quasi-experimental with control groups; pre-post design | Emotional intelligence program. 20x 1hr weekly sessions | EI ability model | Public & private high schools (Spain) | No | 148 (67:81) | Range: 13-16 | EI, aggression, social interaction | General population |
| García-López & Gutiérrez, 2015 | Single-group; pre-post design | Sport education program (Handball classes). 18x 1hr weekly sessions | NR | Primary & secondary schools (Spain) | No | 154 (76:78) | Range: 11-14 | Empathy, assertiveness | General population |
| Kerr, Burke & McKeon, 2011 | Intervention & control group; cross-sectional | Depression information intervention. (Beat the Blues; BTB). 1x ~2hr session | NR | All-male secondary schools; range of socio-economic status (Ireland) | Unclear | 42 (42:0) | 16.4 (NR) | Help-seeking, attitudes towards depression | General population |
| Liddell & Kurpius, 2014* | Experimental; waitlist control. | Masculine identity formation curriculum (The Council for Boys and Young Men). 10x 1-1.5hr weekly sessions | Relational-cultural theory, resiliency principles | Alternative metropolitan high-school (USA) | Yes; Gender-transformative | 22 (22:0) | 16.6 (1.4) | Self-esteem, educational self-efficacy, masculine ideology, identity distress, relational aggression | At-risk; students with behavioural problems |
| Lubans et al., 2015, 2016;  Wade et al., 2018 | Cluster RCT; waitlist control | Physical activity and obesity prevention program (Active Teen Leaders Avoiding Screen-time; ATLAS). 20-week intervention; 20x 1.5hr sport session, 3x 20min interactive seminar, 6x 20min lunch-time activity mentoring sessions, 4x newsletters | SDT | Secondary schools in low-income communities (Australia) | Yes; Gender-sensitive | 361 (361:0) | 12.7 (0.5) | Psychological well-being, motivation regulation, basic psychological needs, aggression | Low socio-economic status (SES), at-risk of obesity males |
| Margalit & Ben-Ari, 2014 | Multi-group with control; pre-post design | Wilderness therapy. Full program; 10x preparation meetings, 1x backpacking trip, 2x closure meetings. Partial program; 10x preparation meetings | Experiential learning | Boarding-schools for at-risk youth (Israel) | NR | 93 (93:0) | Range: 14-16 | Cognitive autonomy, self-efficacy | At-risk; low SES, social or educational difficulties |
| Marsh & Richards, 1988 | Single-group; pre-post design | Outdoor activity program (Outward Bound Bridging Course). 6-week residential program | Achievement motivation theory | High schools; public & private (Australia) | Yes; Gender-sensitive | 66 (66:0) | Range: 13-16 | Academic achievement, self-concept | Low academic achieving males |
| McCabe, Ricciardelli & Karantzas, 2010 | Experimental; pre-post design | Healthy body-image program. 5x 1hr weekly sessions | NR | High schools (Australia) | Yes; Gender-sensitive | 421 (421:0) | 13.0 (0.9) | Negative affect, self-esteem, peer popularity | General population |
| Namy et al., 2015 | Multi-group, cross-sectional | Healthy masculinity promotion program (Young Men Initiative; YMI). Year-long program; 8x 1hr group education sessions, optional residential retreat, ‘Be a Man’ school clubs | Peer-group learning, socialisation. | Vocational high schools (Balkans) | Yes; Gender-transformative | NR; 37 in-depth interviews, 11 focus groups | Range: 14-16 | Manhood attitudes, program feedback | General population |
| O’Dea & Abraham, 2000 | Experimental; pre-post design | Self-esteem & body image promotion program (Everybody’s Different). 9x 50-80min weekly lessons | Educational cooperative learning theory | High schools; public & private, girl’s school & coeducational (Australia) | No | 470 (173:297) | 13.0 (0.6) | Self-esteem, social acceptance, self-worth, anxiety, depression | General population |
| O’Kearney et al., 2006 | Non-randomised control trial | Internet-based CBT depression intervention (MoodGYM). Self-paced interactive internet program, 5x 30-60min online modules. | CBT | High school (Australia) | Unclear | 78 (78:0) | Range: 15-16 | Self-esteem, depression symptoms & attitudes | General population |
| Opper et al., 2014 | Single group; pre-post design | Outdoor adventure program (The Journey). 23-day program | Experiential learning, social cognitive theory, optimal arousal and stretch-zone theories | High school; private, all-male (South Africa) | No | 76 (76:0) | NR, grade 10 | Emotional intelligence | General population |
| Rhodes et al., 2008 | Multi-group; pre-post design | Mentoring program (Big Brothers Big Sisters of America; BBSA). Varied lengths of mentoring | NR | Community program (USA) | No | 959 (599:360) | 12.3 (NR) | Length of mentoring relationship, parent-relationships, satisfaction with mentoring | General population; Applicants to BBSA |
| Ritchie et al., 2014 | Single-group; pre-post design | Outdoor adventure leadership experience. 10-day expedition, 11 separate groups | Outward bound process model | Community program (Canada) | No | 61 (38:23) | 14.6 (NR) | Resilience, SWL, self-esteem, positive and negative affect | General population; aboriginal adolescents |
| Rojiani et al., 2017 | Single-group; pre-post design | Meditation training. 12x weekly seminars. Meditation practice, 1hr 3x/week for 12 weeks | Emotion regulation/coping | University (USA) | No | 77 (41:36) | 20.7 (3.0) | Negative affect, self-compassion mindfulness | General population |
| Sekizaki et al., 2017 | Experimental; pre-post design | Internet-based CBT (iCBT) for mental health promotion. 1x 3hr group CBT education session, 1-month iCBT use | CBT | High school; private, sport-specialist (Japan) | No | 80 (80:0) | 15.8 | Self-efficacy, distress, symptoms of depression & anxiety | Young athletes |
| Shoshani & Steinmetz, 2014 | Non-randomised control trial | Mental health promotion. 15x fortnightly workshops | Positive psychology | Middle-schools (Israel) | No | 1038 (513:525) | 13.7 (0.6) | Self-esteem, self-efficacy, optimism, SWL, distress | General population |
| Sibinga et al., 2013 | RCT | Mindfulness-based stress reduction program. 12x 50-minute weekly mindfulness or ‘healthy topics’ session | Mindfulness-based stress reduction | Urban middle-school (boys with academic potential) (USA) | No | 41 (41:0) | 12.5 (NR) | Coping, psychological functioning, sleep, anxiety, cortisol | Low socio-economic status (SES), |
| Skre et al., 2013 | Non-randomised cluster controlled trial | Mental health literacy program (Mental health for everyone). 3-day intervention; in-class learning and activities | Salutogenesis (positive psychology); health promotion through empowerment | Secondary schools – suburban (Norway) | No | 1070 (577:493) | 14.1-14.3 (0.8-0.9) | Mental health literacy; symptom profile recognition, prejudice beliefs | General population |
| Smith, 2012 | Single-group, pre-post design | Rite of passage program (The Rite Journey; TRJ). Year-long in-class teaching, 7x ‘ceremonies’ including 1x 24-hour solo wilderness camp | Five-C’s model; consciousness, connection, communication, celebration & challenge | Secondary school; all-boys (New Zealand) | Yes; Gender-transformative | 8 (8:0) | Range: 13-14 | Attitudes about manhood, program feedback | General population |
| Stanford & McCabe, 2005 | Non-randomised control trial | Body-image promotion program. 2x 1hr sessions group learning and activities. | NR | Metropolitan secondary schools (Australia) | Yes; Gender-sensitive | 121 (121:0) | 12.2 (0.4) | Self-esteem, negative affect, body image | General population |
| Switzer et al., 1995 | Quasi-experimental with control group; pre-post design | Helper program (Early Adolescent Helper Program). Compulsory community—based service and weekly seminars, e.g. tutoring or volunteering. Weekly 1h volunteering for 7 months & weekly seminar | Social-learning theory | Metropolitan middle-school (USA) | No | 171 (63:108) | Range: 11-13 | Self-image, school & community commitment, altruism, depressive affect, problem behaviours | General population |
| Taylor, Gillies & Ashman, 2009 | Counterbalanced experimental design | 3x intervention; explanatory style cognitive training, conflict resolution & exercise. 8x 1hr explanatory sessions twice weekly, 6x 1hr conflict resolution role-play sessions over 1-month, 4 week self-regulated exercise regime | Hopelessness theory | Metropolitan private boys school (Australia) | No | 31 (31:0) | 12.6 (11.0) | Well-being, coping, depression symptoms | At risk; depression symptoms present |

*Note.* Not reported (NR), *dissertation.
