# Supplementary material for: Developing young men’s wellbeing through community and school-based programs: A systematic review
Source: PLoS One. 2019 May 20;14(5):e0216955. doi: 10.1371/journal.pone.0216955 (PMC6527294; doi:10.1371/journal.pone.0216955)
Supplement: S2 Table — (DOCX) [file pone.0216955.s003.docx]

**S2 Table. Article Results Summary**

| **Authors** | **Assessment Schedule** | **Main Findings** |
| --- | --- | --- |
| Ashton et al., 2017 | Baseline, post-program (3 months post baseline) | The intervention group showed a significant increase in QoL enjoyment and satisfaction (*MD* = 6.0, 95% CI = 3.3 – 8.7) and a significant decrease in psychological distress (*MD* = -2.6, 95% CI = -3.8 – -1.3). There were no differences between the intervention and control group in these measures. |
| Bademci, Karadayi & de Zulueta, 2015 | Continuous reporting after each session, qualitative interview post program | Participants reported reduced aggression and increased self-esteem and motivation (“*I do not fight”; “I was embarrassed but now I’m not”; “I have plans for the future”)*. 16.67% of participants returned to the streets while the remaining participants continued with the program (35.41%), joined school or other training courses (21.25%), or re-joined their families (16.67%). |
| Bannink et al., 2014a & 2014b | Baseline, post-program (4 months post baseline) | For the whole sample (males & females), the intervention group (E-Health4Uth) showed improved health-related QoL compared to the control group (*B* = 2.79, 95% CI = 0.72 - 4.87). Additionally, the second intervention group (E-Health4Uth + consultation) demonstrated enhanced mental health status comparative to controls (*B* = -0.60, 95% CI = -1.17 – -0.04). Girls were more satisfied with the program than boys (*p* < .01), found the messages more credible (*p* = .03) and easier to understand (*p* < .01). Girls were more likely to have discussed their health messages with their peers than boys (*p* = .04). |
| Bluth, Robertson & Girdler, 2017 | Baseline, post-program (6 weeks post baseline) | Males reported no changes post intervention for perceived stress, life satisfaction or self-compassion. Males showed strong increased positive affect (*d* = 1.33, 95% CI = 0.42 – 2.24) and a trending decrease in negative affect (*d* = -0.25, 95% CI = -0.47 – 0.03) whereas females showed no changes. Females were more engaged in the mindfulness intervention compared to males. |
| Broadbent & Papadopoulos, 2014 | Semi-structured interview up to 24 months post program, survey ~ 6 months post program | Participants most commonly reported aggression, stress and being withdrawn as signs of someone ‘doing it tough’. Roughly 80% of participants indicated the program helped them understand how to identify and seek help for problems in themselves and others. Approximately 50% of participants indicated they felt comfortable reaching out to a care worker if they were having problems. 17 participants sought help after attending the workshop. Reported behavioural change was relatively low (35%), with 11% citing increased awareness of others and 8% talking more to others. |
| Burns et al., 2010 & Shandley et al., 2010 | Baseline, post-program, 2 month follow-up | 52% of 10,542 new members were male, suggesting males were interested in the online program. Males did not report any significant changes in psychological distress, SWL or problem solving. There was also substantial drop-out of male participants (66/88), indicating successful appeal to young men but failure to maintain engagement. Males (and females) reported significantly higher likelihood to seek psychological help post-program and at follow-up (*F*_(1,264)_ = 18.04, *p* < .01). Females displayed lower mental health stigma, reporting higher willingness to befriend someone with depression than males post-program (*F*_(1, 264)_ = 4.00, *p* = .04) and follow-up (*F*_(1, 264)_ = 4.74, *p* = .03). |
| Campbell-Heider, Tuttle & Knapp, 2009 | Baseline, post-program, 12 month follow-up | Boys in the Teen Club group had fewer mental health problems at follow-up (*M* = 4.2) compared to baseline (*M* = 6.0). Boys in the PALS group showed fewer problems in mental health (*M* = 3.67, 8.33) peer relations (*M* = 1.67, 4.0) and aggression (*M* = 3.33, 7.33) at follow-up compared to baseline respectively. |
| Castillo et al., 2013 | Baseline, post-program (2 years post baseline) | Post-program differences indicated that the intervention group (males and females) showed less physical aggression (*F*_(1, 581)_ = 7.67, *p* <.01) verbal aggression (*F*_(1, 581)_ = 5.82, *p* = .02), hostility (*F*_(1, 581)_ = 18.08, *p* <.01) and anger (*F*_(1, 581)_ = 7.15, *p* <.01) compared to the control group. For empathy, males in the intervention group showed significantly lower levels of fantasy (*F*_(1, 581)_ = 7.41, *p* <.01), personal distress (*F*_(1, 581)_ = 5.53, *p* =.02) and higher empathic concern (*F*_(1, 581)_ = 4.86, *p* =.03) compared to control group males. |
| Crooks et al., 2017 | Wave 1, baseline; Wave 2, 1 year follow-up; Wave 3, 2 year follow-up | At Wave 3, the 2-year mentoring intervention group had greater positive mental health (*t*_(100)_ = -2.59, *p* =.01) and stronger cultural identity (*t*_(101)_ = -2.67, *p* < .01) compared to the 1-year or no mentoring groups. These results were maintained after controlling for gender. However, simple slopes analysis found that the association between 2-year mentoring, mental health (*b* = 11.23, *p* = .04) and identity (*b* = 3.95, *p* = .02) was only significant for females. Qualitative findings suggest positive intra and interpersonal outcomes for males and females regarding culture, identity, confidence and leadership. |
| Eather, Morgan & Lubans, 2016 | Baseline, post-program (8 weeks post baseline) | There were no significant direct treatment effects on self-esteem or psychological difficulties for males or females. |
| Edwards, van de Mortel & Stevens, 2017 | Baseline (demographics only), mid-point (5 weeks), post-program (9 weeks). | Qualitative interviews post-program indicated that males had reduced anger (*“now I am getting less reactive” “I just kept eye contact and walked away”*). Males reported enjoyment of the RWP, specifically they enjoyed the action-oriented nature, games and competitions and learning by doing. |
| Eteokleous, 2011 | Baseline, post-program (14-15 months post baseline) | Baseline comparisons between genders showed that girls* had higher interest in diversity of contact (DC). This difference was no longer evident post-program. ***unclear** |
| Fuller et al., 2013 | Mid-program (week 12), post-program (week 24) | From qualitative interviews, competence was coded the most (114 times), followed by confidence (63), character (56) connection (36), contribution (15) and caring (8). Young boys noted changes in their social, cognitive, physical and nutritional competence (“*I’m not demanding people”, “I learned that I can do more stuff”*). |
| Garaigordobil & Pena-Sarrionandia, 2015 | Baseline, post-program (1 school year post baseline), 1 year follow-up | At post-program and follow-up, the intervention group showed a significant increase in emotional attention, emotional clarity and emotional repair compared to the control group. Gender differences were found at baseline, whereby females had higher emotional attention, assertive social interaction, state-anger, identification of causes for negative feelings and less external control of anger compared to males. Pre-test to post-test changes showed that males improved significantly more than girls in emotional attention (*F*_(1, 81)_ = 4.03, *p* < .05), emotional clarity (*F*_(1, 81)_ = 4.33, *p* = .04) and assertive social-conflict resolution strategy (*F*_(1, 81)_ = 4.37, *p* = .04). |
| García-López & Gutiérrez, 2015 | Baseline, mid-point (unclear), post-program (~18-weeks post baseline) | Males displayed increased assertiveness from pre-test (*M* = -15.48) to post-test (*M* = -5.03). There were no significant differences in empathy by gender. |
| Kerr, Burke & McKeon, 2011 | Post-program | There were no differences between intervention and control groups on attitudes about mental illness. Differences arose when boys were compared by socio-economic status whereby those in the lower socio-economic group held more negative beliefs about mental illness (*F*_(2, 39)_ = 7.20, *p* < .01). |
| Liddell & Kurpius, 2014* | Baseline, post-program (10 weeks post baseline) | Boys in the intervention group showed increased school self-efficacy (*F*_(1, 17)_ = 5.50, *p* = .03) and future self-efficacy (*F*_(1, 16)_ = 11.92, *p* <.01) compared to the control group. No differences were found in masculine ideology, identity distress or relational aggression following the intervention. At pre-test, high stereotypical masculine ideology was associated with higher relational aggression (*r* = .40, *p* < .05) and lower self-esteem (*r* = -.43, *p* < .05). |
| Lubans et al., 2015 & 2016 Wade et al., 2018 | Baseline, post-program (8 months post baseline) | There was a significant intervention effect on psychological well-being (unstandardised regression coefficient *C* = 1.40, *p* =.03) whereby boys in the ATLAS group showed an increase in well-being. This effect was mediated by autonomy needs satisfaction (*AB* = .026) and recreational screen time (*AB* = .044). There were no interaction effects for aggression, however a significant reduction in screen-time mediated post-test reduction in aggression (*AB* = -.021). |
| Margalit & Ben-Ari, 2014 | Wave 1, baseline; Wave 2, post-program (6 months post baseline); Wave 3, 5 month follow-up | Baseline results indicated no significant differences between intervention, partial-intervention (& re-allocated) and control groups on measures of cognitive autonomy and self-efficacy. Within-groups, boys in the intervention and partial-intervention groups showed increased cognitive autonomy and self-efficacy in waves 2 and 3 compared to wave 1. No changes were found in the control group. Between groups, a significant difference was found in self-efficacy (*η^2^* = 0.66) and cognitive autonomy (*η^2^* = 0.68), whereby the intervention group displayed higher scores compared to all other groups. |
| Marsh & Richards, 1988 | T1, baseline (6 weeks pre-intervention); T2, beginning of program (day 1); T3, post-program (6 weeks post T2). | Boys displayed an increase in total academic self-concept (*M* = 3.25, 3.82) and academic self-esteem (*M* = 3.93, 5.24) between T2 and T3 respectively. Overall self-esteem scores showed a small increase between T2 (*M* = 33.65) and T3 (*M* = 38.56). Mathematic and reading test scores improved significantly from T2 to T3. |
| McCabe, Ricciardelli & Karantzas, 2010 | T1, baseline; T2, post-program (~6 weeks post T1); T3, 3 month follow-up; T4, 6 month follow-up; T5, 12 month follow-up | There were no differences in outcomes at T1 between schools. No significant intervention effects were found in negative affect, self-esteem or peer popularity. Boys with lower body satisfaction showed a reduction in negative affect after the intervention compared to controls (*β* = .40, *p* <.05). |
| Namy et al., 2015 | Post-program | Participants reported changed thinking about masculine ideals and what it means to be a man (*“there should be no difference between a man and a woman”, “I kept thinking, who am I? What kind of man am I?”, “I think ‘Be a Man’ has changed my opinion completely”*). Predominant themes from focus groups and interviews were personal reflection, experience-based learning, connection with facilitators, new peer groups and aspirational messaging. |
| O’Dea & Abraham, 2000 | Baseline, post-program (3 months post baseline), 12 month follow-up | Total sample (males and females) showed improved self-esteem whereby social acceptance and physical appearance became less important. Male and female participants at-risk of eating disorder (high trait anxiety and low self-esteem) showed a significant decrease in body dissatisfaction scores post intervention (*F*_(1, 111)_ = 4.8, *p* <.05). |
| O’Kearney et al., 2006 | Baseline, post-program (~2 months post baseline), 16 week follow-up | Participants that completed ≥ 3 online modules demonstrated small improvements in attributional style (*ES* = 0.17), self-esteem (*ES* = 0.16) and depressive symptoms (*ES* = 0.34) for post-program measures. Only self-esteem remained significant at follow-up (*ES* = 0.21). |
| Opper et al., 2014 | Baseline, post-program (~4 weeks post baseline), 3 month follow-up | Post-program intervention effects indicated increased overall emotional intelligence (partial *η^2^* = .39), intrapersonal skills (partial *η^2^* = .24), adaptability (partial *η^2^* = .30) and mood subscales (partial *η^2^* = .17). Effect sizes decreased yet remained significant at follow-up. No differences were found for interpersonal or stress management subscales at follow-up. |
| Rhodes et al., 2008 | Baseline, 18 month follow-up | On average, girls remained in mentoring relationships one month longer than boys (*t* = -2.04, *p* =.04). Boys in short-term mentoring (1-6 months) were less satisfied than boys in medium-term mentoring (7-12 months) and long-term mentoring (13-18 months). |
| Ritchie et al., 2014 | T1, baseline (1 day pre-program); T2, 1 month follow-up; T3, 1 year follow-up | There was a short-term increase on male’s resilience between T1 (*M* = 71.30) and T2 (*M* = ~76.20), Males aged 12-14 and 15-18 returned to baseline resilience at T3. For the entire group (males and females), increases from T1 to T2 were found for mental health, physical health, self-esteem, flourishing, emotional balance and SWL. Stable increases from T1 to T3 were found for mental health, balance of emotion and SWL. |
| Rojiani et al., 2017 | Baseline, post-program (~12 weeks post baseline) | A time x gender interaction for negative affect indicated that females showed significantly decreased negative affect after the program, whereas males did not (*F*_(1, 76)_ = 9.00, *p* <.01). Both males and females showed a significant increase in self-compassion after the mindfulness program (male *MD* = 8.01, female *MD* = 13.11, *p* <.01). Decrease in pre-post negative affect in males was correlated with increased ability to describe emotions (*r* = -0.38). |
| Sekizaki et al., 2017 | Baseline, post-program (4 weeks post baseline) | A significant time x group interaction indicated that the iCBT intervention group showed a non-significant decrease in distress whereas the control group showed a significant increase (*F*_(1, 78)_ = 7.28, *p* <.01). |
| Shoshani & Steinmetz, 2014 | T1, baseline; T2, post-program (9 months post baseline); T3, follow-up (6 months post program); T4, follow-up (12 months post program) | At baseline, males had higher anxiety (*d* = 0.32), lower scores on the Global Severity Index (*d* = 0.19), fewer depressive symptoms (*d* = 0.25) and lower interpersonal sensitivity (*d* = 0.38) than females. Additionally at baseline, males displayed higher self-esteem and lower self-efficacy, optimism and SWL than females. After the intervention, males showed a greater increase in self-efficacy and a smaller increase in general distress, interpersonal sensitivity and optimism compared to females. |
| Sibinga et al., 2013 | Baseline, post-program (within 2 weeks post intervention), 3 month follow-up | The mindfulness intervention group showed a significant reduction in anxiety (*β* = -3.74, *p* =.01) and rumination (*β* = -5.32, *p* =.02). There were no differences between intervention and control groups for sleep measures. In the intervention group, mindfulness was associated with lower anger temperament (*p* < .02) and anger reactivity (*p* =.05). |
| Skre et al., 2013 | Baseline, 2 month follow-up | At baseline, females were significantly more likely to have correctly named anxiety, depression and eating disorder, but not schizophrenia, from a symptom profile compared to males. After the mental health literacy program, the intervention group showed a significant increase in correct identification for all 4 disorders profiles, however, symptom profile recognition remained higher in females than males (*β* = -.42). Despite a reduction of prejudiced beliefs in the intervention group, prejudiced beliefs were significantly higher in males than females (*β* = .43). Males were more likely than females to suggest primary health care (Exp(B) = 0.71) and specialist health care (Exp(B) = 1.26) as options for mental health help-seeking. |
| Smith, 2012 | Baseline, post-program (~12 months post baseline) | At baseline, boys attitudes towards ‘manhood’ included sentiments of power and intelligence (“*pretty much doing whatever he likes whenever he wants”, “get a job, get your licence, go to uni”).* After the program, 3/8 boys believed the program had changed their view on ‘being a man’ (*“I feel nicer towards other people – not being so mean – forgiving people”, “I’ve got to step it up a bit – get on with my work, stop messing around”*) and 3/8 believed it had not. The most common theme was improved relationships between students and staff. |
| Stanford & McCabe, 2005 | T1, baseline; T2, post-program (2 weeks post baseline), T3, follow-up (1 month post T2); T4, follow-up (3 months post T2) | Over the four time periods, boys in the intervention group showed increased self-esteem (*F*_(2.4, 167.6)_ = 3.2, *p* <.05) and decreased negative affect (*F*_(2.8, 196.2)_ = 2.64, *p* <.05), whereas the control group demonstrated decreased self-esteem and varying negative affect. |
| Switzer et al., 1995 | Baseline, post-program (~8 months post baseline) | At baseline, boys had higher self-esteem, school problem behaviour and involvement in school and community activities, and lower perceived altruism compared to girls. Boys in the intervention group showed significant improvements for self-esteem (*F* = 9.60, *p* < .05) and school involvement (*F* = 9.27, *p* = .01), as well as significantly reduced depressive affect (*F* = 6.03, *p* < .01) and school problem behaviour (*F* = 13.13, *p* < .01) comparative to girls and control boys. |
| Taylor, Gillies & Ashman, 2009 | Baseline, post-program 1 (4 weeks post baseline), post-program 2, (8 weeks post baseline) post-program 3 (~10 weeks post baseline) | The intervention group showed significant decrease in worry (*F* = 23.76, *p* <.01) and self-blame (*F* = 15.01, *p* <.01) coping strategies, decreased depressive symptoms (*F*_(3, 72)_ = 3.15, *p* <.05) and increased optimism (*F*_(1, 24)_ = 10.91, *p* <.05). Similar patterns were found for parental perceptions whereby parents perceived lower aggression (*F* = 11.29, *p* <.01) and improved well-being (*F*_(2.2, 36.91)_ = 6.78, *p* <.01) in their sons. Student’s reported the greatest improvement for depressive symptoms after the exercise intervention. |
| *dissertation |  |  |
