# Supplementary material for: Developing young men’s wellbeing through community and school-based programs: A systematic review
Source: PLoS One. 2019 May 20;14(5):e0216955. doi: 10.1371/journal.pone.0216955 (PMC6527294; doi:10.1371/journal.pone.0216955)

|  | Bademci et al. 2015 | Bluth et al., 2017 | Broadbent & Papadopoulos, 2014 | Burns et al., 2010 | Campbell-Heider et al., 2009 | Castillo et al., 2013 | Crooks et al., 2017 | Edwards et al., 2017 | Eteokleous et al., 2011 | Fuller et al., 2013 | Garaigordobil & Pena-Sarrionandia, 2015 | García-López & Gutiérrez, 2015 | Kerr et al., 2011 | Liddell & Kurpius, 2014 | Margalit & Ben-Ari, 2014 | Marsh & Richards, 1988 | McCabe et al., 2010 | Namy et al., 2015 | O’Dea & Abraham, 2000 | O’Kearney et al., 2006 | Opper et al., 2014 | Rhodes et al., 2008 | Ritchie et al., 2014 | Rojiani et al., 2017 | Sekizaki et al., 2017 | Shandley et al., 2010 | Shoshani & Steinmetz, 2014 | Skre et al., 2013 | Smith, 2012 | Stanford & McCabe, 2005 | Switzer et al., 1995 | Taylor et al., 2009 | Total ✓/32 |
| --- | --- | --- | --- | --- | --- | --- | --- | --- | --- | --- | --- | --- | --- | --- | --- | --- | --- | --- | --- | --- | --- | --- | --- | --- | --- | --- | --- | --- | --- | --- | --- | --- | --- |
| Q1 | ✓ | ✓ | ✓ | ✓ | ✓ | ✓ | ✓ | ✓ | ✓ | ✓ | ✓ | ✓ | ✓ | ✓ | ✓ | ✓ | ✓ | ✓ | ✓ | ✓ | ✓ | ✓ | ✓ | ✓ | ✓ | ✓ | ✓ | ✓ | ✓ | ✓ | ✓ | ✓ | 32/32 100% |
| Q2 | ✓ | ✓ | N/A | ✓ | ✓ | ✓ | ✓ | ✓ | ✓ | ✓ | ✓ | ✓ | × | ✓ | × | × | ✓ | N/A | ✓ | ✓ | ✓ | ✓ | ✓ | ✓ | ✓ | ✓ | ✓ | ✓ | ✓ | ✓ | ✓ | ✓ | 27/30 90% |
| Q3 | ✓ | ✓ | N/A | ✓ | ✓ | ✓ | ✓ | ✓ | ✓ | ✓ | ✓ | ✓ | ✓ | ✓ | ✓ | ✓ | ✓ | N/A | ✓ | ✓ | ✓ | ✓ | ✓ | ✓ | ✓ | ✓ | ✓ | ✓ | ✓ | ✓ | ✓ | ✓ | 30/30 100% |
| Q4 | × | × | × | × | × | ✓ | ✓ | × | × | × | ✓ | × | ✓ | ✓ | ✓ | × | ✓ | × | ✓ | ✓ | × | ✓ | ✓ | × | ✓ | × | ✓ | ✓ | × | ✓ | ✓ | ✓ | 17/32 53% |
| Q5 | ✓ | ✓ | × | ✓ | ✓ | ✓ | ✓ | × | ✓ | × | ✓ | ✓ | × | ✓ | ✓ | ✓ | ✓ | × | ✓ | ✓ | ✓ | ✓ | ✓ | ✓ | ✓ | ✓ | ✓ | ✓ | ✓ | ✓ | ✓ | ✓ | 27/32 84% |
| Q6 | ✓ | ✓ | N/A | × | ✓ | ✓ | ✓ | ✓ | ✓ | ✓ | ✓ | ✓ | N/A | ✓ | ✓ | ✓ | ✓ | N/A | ✓ | ✓ | ✓ | ✓ | × | × | ✓ | × | ✓ | ✓ | ✓ | ? | × | ? | 22/29 76% |
| Q7 | ✓ | ✓ | N/A | ✓ | ✓ | ✓ | × | ✓ | ✓ | ✓ | ✓ | ✓ | ✓ | ✓ | ✓ | × | ✓ | N/A | ✓ | ✓ | ✓ | ✓ | ✓ | ✓ | ✓ | ✓ | ✓ | × | ✓ | ✓ | ✓ | ✓ | 27/30 90% |
| Q8 | ✓ | ✓ | ✓ | ✓ | ✓ | ✓ | ✓ | ✓ | ✓ | ✓ | ✓ | ✓ | ✓ | ✓ | ✓ | ✓ | ✓ | ✓ | ✓ | ✓ | ✓ | ✓ | ✓ | ✓ | ✓ | ✓ | ✓ | ✓ | ✓ | ✓ | ✓ | ✓ | 32/32 100% |
| Q9 | N/A | ✓ | ✓ | ✓ | ✓ | ✓ | ✓ | N/A | ✓ | N/A | ✓ | ✓ | ✓ | ✓ | ✓ | ✓ | ✓ | N/A | ✓ | ✓ | ✓ | ✓ | ✓ | ✓ | ✓ | ✓ | ✓ | ✓ | N/A | ✓ | ✓ | ✓ | 27/27 100% |
| Total  ✓, % | 7/8  88% | 8/9  89% | 3/5  60% | 7/9  78% | 8/9  89% | 9/9  100% | 8/9  89% | 6/8  75% | 8/9  89% | 6/8  75% | 9/9  100% | 8/9  89% | 6/8  75% | 9/9  100% | 8/9  89% | 6/9  67% | 9/9  100% | 2/4  50% | 9/9  100% | 9/9  100% | 8/9  89% | 9/9  100% | 8/9  89% | 7/9  78% | 9/9  100% | 7/9  78% | 9/9  100% | 8/9  89% | 7/8  88% | 8/9  89% | 8/9  89% | 8/9  89% |  |

**S3a Table. Quasi-experimental quality appraisal – Joanna Briggs Assessment**

*Note.* Not reported (?).

**S3b Table.** **RCT quality appraisal – Joanna Briggs Assessment**

|  | Ashton et al., 2017 | Bannink et al., 2014a | Bannink et al., 2014b | Eather et al., 2016 | Lubans et al., 2015 | Lubans et al., 2016 | Sibinga et al., 2013 | Wade et al., 2018 | Total ✓/8 |
| --- | --- | --- | --- | --- | --- | --- | --- | --- | --- |
| Q1 | ✓ | ✓ | ✓ | ✓ | ✓ | ✓ | ✓ | ✓ | 8/8  100% |
| Q2 | ✓ | ✓ | ✓ | ✓ | ✓ | ✓ | ✓ | ✓ | 8/8  100% |
| Q3 | ✓ | ✓ | ✓ | ✓ | ✓ | ✓ | ✓ | ✓ | 8/8  100% |
| Q4 | × | ? | ? | × | × | × | × | × | 0/8  0% |
| Q5 | × | ? | ? | × | × | × | × | × | 0/8  0% |
| Q6 | ✓ | ? | ? | ✓ | × | × | × | × | 2/8  25% |
| Q7 | ✓ | ✓ | ✓ | ✓ | ✓ | ✓ | ✓ | ✓ | 8/8  100% |
| Q8 | ✓ | ✓ | ✓ | ✓ | ✓ | ✓ | ✓ | ✓ | 8/8  100% |
| Q9 | ✓ | ✓ | ✓ | ✓ | ✓ | ✓ | ✓ | ✓ | 8/8  100% |
| Q10 | ✓ | ✓ | ✓ | ✓ | ✓ | ✓ | ✓ | ✓ | 8/8  100% |
| Q11 | ✓ | ✓ | ✓ | ✓ | ✓ | ✓ | ✓ | ✓ | 8/8  100% |
| Q12 | ✓ | ✓ | ✓ | ✓ | ✓ | ✓ | ✓ | ✓ | 8/8  100% |
| Q13 | ✓ | ✓ | ✓ | ✓ | ✓ | ✓ | ✓ | ✓ | 8/8  100% |
| Total  ✓, % | 11/13  85% | 10/13  77% | 10/13  77% | 11/13  85% | 10/13  77% | 10/13  77% | 10/13  77% | 10/13  77% |  |

*Note.* Not reported (?).


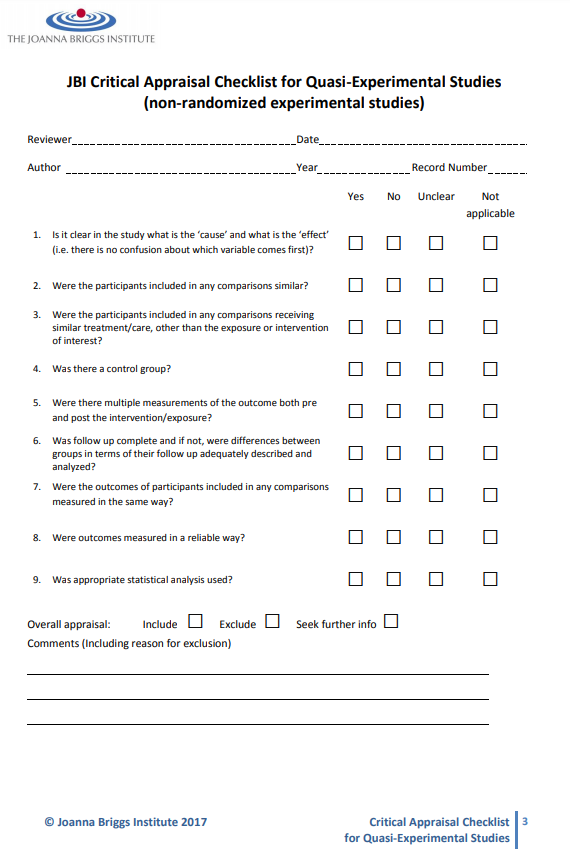


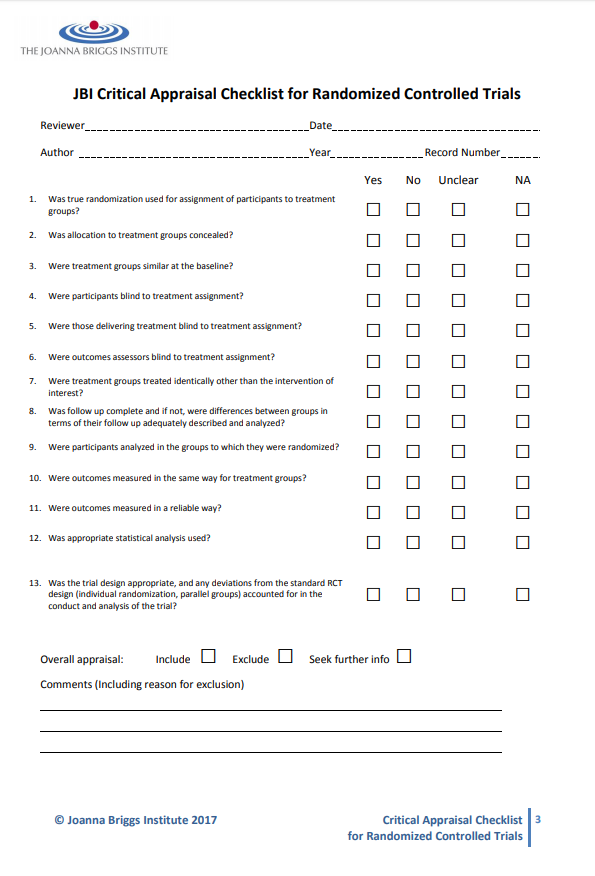

Supplement: S3 Table — a. Quasi-experimental quality appraisal–Joanna Briggs Assessment; b. RCT quality appraisal–Joanna Briggs Assessment. (DOCX) [file pone.0216955.s004.docx]
